# The role of a midwife during perinatal anorexia nervosa care: A narrative review

**DOI:** 10.18332/ejm/236730

**Published:** 2026-10-09

**Authors:** Jade Steppe, Astrid Claerbout, Maja Lopez Hartmann, Mieke Embo, Peggy Tahir, Kathleen Van Royen, Jente Bontinck, Joni Gilissen

**Affiliations:** 1 Research Centre Care in Connection, Karel de Grote University of Applied Sciences and Arts, Antwerp, Belgium; 2 Research and Innovation Centre Health and Care, Artevelde University of Applied Sciences, Ghent, Belgium; 3 Department of Educational Studies, Faculty of Psychology and Educational Sciences, Ghent University, Ghent, Belgium; 4 Department of Family Medicine and Population Health, University of Antwerp, Antwerp, Belgium; 5 Atlantic Fellows for Equity in Brain Health, Atlantic Institute, Oxford, United Kingdom; 6 UCSF Library, University of California, San Francisco, United States; 7 Department of Family Medicine and Chronic Care, Vrije Universiteit Brussel, Brussels, Belgium; 8 Department Master in Nursing and Midwifery, Advanced Midwifery Practice Faculty of Medicine and Health Sciences, University of Antwerp, Antwerp, Belgium

**Keywords:** pregnancy, midwifery, anorexia nervosa, perinatal care, eating disorder

## Abstract

Anorexia nervosa primarily affects women of reproductive age, with prevalence significantly increasing during pregnancy. Identifying optimal ways to support this population during the perinatal period is essential. This narrative review examines the role of midwives in perinatal care of women with anorexia nervosa, the challenges they encounter, and the effectiveness of midwifery-led care in this context. As part of this narrative review, a literature search was conducted across PubMed, CINAHL, Embase, Web of Science, and PsycINFO. Reference lists of included studies were screened for additional sources. Two researchers independently performed screening and data extraction using a structured form. Findings were thematically analyzed and reported narratively. Sixteen articles published between 2000 and 2022, primarily from the United Kingdom and the United States, were included. Four key themes related to the midwife's role were identified: early and continuous screening, continuity of care, building a trusted relationship, and nutrition counseling and breastfeeding support. Midwives frequently overlook lifetime eating disorder diagnoses, underscoring the need for routine screening. Challenges arise from limited knowledge and awareness among midwifery professionals. Midwives might have an added value for women with anorexia nervosa during perinatal time. Early screening, individualized support, and strong interprofessional collaboration – including timely specialist referral – are essential. Education and training are key to improving identification, management, and communication. A midwifery-led care model holds promise for fostering the trusting relationships this population requires. The absence of clinical guidelines highlights an urgent need for further research and guideline development.

## INTRODUCTION

Anorexia nervosa (AN) is a severe psychiatric condition causing medical complications, leading to the well-known high risk of death^1,2^. It is characterized by self-starvation, marked weight loss, and malnutrition, driven by severe body image distortion and intense fear of weight gain, leading to dietary restriction, weight-loss behaviors, and/or excessive physical activity, with severe cognitive and emotional impairment^3^. In women, it can lead to numerous female-specific medical complications, such as amenorrhea due to hormonal disorders^4^. The DSM-5 classifies severity based on BMI into four categories (mild, moderate, severe, and extreme) and distinguishes between the restrictive and purging subtypes. Treatment failure and relapse are common, eating disorders are frequently undertreated, and comorbidities such as anxiety, depression, and substance abuse are often only partially addressed^5^. Although the prevalence of AN varies across ages, sexes, races, and ethnicities^6^, estimates report between 1% and 4% of women developing AN during their lifetime^7^. However, epidemiological research on eating disorders is quite complex due to the lack of uniformity of studies and the modification of diagnostic criteria over time^8^.

Anorexia nervosa mainly affects girls and young adult women who are often in their reproductive age, with an increasing trend of incidence of onset at younger ages^7^. Although it was assumed that women with anorexia had little or no chance to conceive, recently it has become clear that pregnancy can occur even with amenorrhea or irregular menstruation, and this can be potentially dangerous for women and their fetus^1^. The prevalence of eating disorders during pregnancy ranges between 0.5–10.6%, with a pooled estimate of 4.3%, and shows a statistically significant increase in anorexia nervosa and binge eating disorder compared to pre-pregnancy^9^. Prevalence estimates specific to anorexia nervosa in pregnancy are lower, between 0.05% and 0.50%^10^. Half of all pregnant women with an eating disorder experience anxiety, and one-third experience depression during their pregnancy^9^.

Despite the well-documented risks of AN during pregnancy for both mother and child, midwives – who are often the primary and most continuous point of contact during the perinatal period – currently lack specific guidance on how to identify and support this population. Existing literature on managing AN in pregnancy has been written primarily for gynecologists and specialist providers^10^, leaving a significant gap in midwifery-focused evidence. As midwife-led care models expand globally^11^ and midwives take on increasingly broad responsibilities, it is essential to synthesize what is known about their potential role in caring for women with AN during the perinatal period. The perinatal period also represents a critical window of opportunity: women are in frequent contact with healthcare services, yet AN often goes undetected due to non-disclosure and insufficient awareness among midwives.

We need more information on what midwives need to provide safe, effective, and sensitive perinatal care to women with anorexia nervosa. This review aimed to examine what is known in academic literature about the (potential) role of a midwife during the perinatal period in women with (a history of) anorexia nervosa; what are the challenges (encountered by patients, midwives, or other healthcare providers) regarding perinatal care that can be relevant for a midwife in providing perinatal care to these women; and what is known about the effectiveness of perinatal care for women with anorexia nervosa when provided by a midwife.

We conducted a narrative review following the five-stage JBI methodology. We registered the protocol on the Open Science Framework (ref: DOI 10.17605/OSF.IO/FEKRP). We conducted the review between October 2023 and the end of September 2024. We followed the PRISMA checklist for reporting.

Academic literature was searched to identify peer-reviewed publications. The inclusion criteria (Table 1)^1,12^ required literature published from 2000 onward, in English, Dutch, or French, focused on midwives and patients diagnosed with anorexia nervosa, as per DSM-5 criteria, encompassing the recovery (including the relapse phase), passive phase, and active phase, which includes subsyndromal, full syndrome, persistent illness, partial remission, and full remission. The year 2000 was chosen as the start date to ensure relevance to contemporary diagnostic criteria, as the DSM-IV (published in 1994) introduced significant revisions to the diagnostic criteria for anorexia nervosa that became widely adopted in clinical and research practice by 2000. Studies were excluded if they focused on other healthcare providers, mixed populations without separate reporting on midwives, or eating disorders other than anorexia nervosa. The review included primary studies, reviews, and various types of publications, with no restrictions on study design or care setting. Midwifery-specific search terms were deliberately omitted from the search strategy, as the aim was to capture all literature relevant to perinatal care in women with anorexia nervosa that might inform the midwife’s role, even where midwives were not explicitly named.

**Table 1:** Eligibility criteria applied during study selection for a narrative review on the role of midwives in perinatal anorexia nervosa care

	*Inclusion criteria(combined with AND)*	*Exclusion criteria (combined with OR)*
**Publication date**	From 2000 until date of search	Literature published before 1 January 2000
**Language**	English, Dutch, French	
**Population**	Humans; focus on midwives (all types of midwives who completed the midwifery education program with the essential competencies and framework of the ICM global standards for midwifery education, recognized in the country where it is located).	Animals; papers/studies focusing on other healthcare providers or focusing on/including mixed populations of healthcare providers (including midwives) but where findings are not reported separately
**Disease**	Anorexia nervosa defined as: ‘1) Restriction of energy intake relative to requirements, leading to a significantly low body weight in the context of age, sex, developmental trajectory, and physical health. Significantly low weight is defined as a weight that is less than minimally normal or, for children and adolescents, less than that minimally expected; 2) Intense fear of gaining weight or of becoming fat, or persistent behavior that interferes with weight gain, even though at a significantly low weight; and 3) Disturbance in the way in which one’s body weight or shape is experienced, undue influence of body weight or shape on self-evaluation, or persistent lack of recognition of the seriousness of the current low body weight.’ (DSM-5)^1^ All types of anorexia nervosa (i.e. restricting type and binge-eating type)	Eating disorders other than AN, i.e. bulimia nervosa (BN), binge eating disorder (BED), other specified feeding and eating disorder (OSFED), Pica, rumination disorder, avoidant/restrictive food intake disorder (ARFID), unspecified feeding or eating disorder (UFED), other, i.e. muscle dysmorphia, orthorexia nervosa (ON) proposed criteria. Studies/papers focusing on/including mixed diseases and when findings are not reported categorized by disease group.
**Type of publication**	Primary study or review (see study design), book or book chapter, editorial or discussion article, individual case report, evaluation of local program/audit, report of consensus meeting(s), questionnaire (validation), or training session, and PhD thesis	Opinion papers and study protocols.
**Subject/intervention**	Involvement of midwives can range from preventative measures, the promotion of normal birth, the detection of complications in mother and child, the accessing of medical care or other appropriate assistance and the carrying out of emergency measures; health counselling and education^12^.	
**Study design**	Literature reviews and research using any methodology or method (i.e. data are collected and analyzed), both qualitative and quantitative are included.	No restrictions on the types of study design.
**Setting**	Any setting a midwife may practice.

- A ‘midwife’ was defined as someone who has completed the midwifery education program with the essential competencies and framework of the ICM global standards for midwifery education, recognized in the country where it is located^13^.
- The ‘care provided by a midwife’ could involve primary care, well-woman care/gynecology, antenatal, intrapartum, newborn, and postpartum care.

We included publications reporting on women with anorexia nervosa during the perinatal period, encompassing the recovery (including the relapse phase), passive phase, and active phase, which includes subsyndromal, full syndrome, persistent illness, partial remission, and full remission. Remission was defined as being asymptomatic for at least one year. We included publications focusing on the perinatal period, from preconception, through the prenatal period, labor and birth, and up to one year postpartum.

A comprehensive search strategy (Supplementary file Table 1) was developed in consultation with a librarian (PT) and ran in five electronic databases: MEDLINE (PubMed), Web of Science, Embase, CINAHL and PsycINFO. Reference lists of included publications were screened for additional studies. All potentially relevant articles were collected and imported into the reference manager software, Zotero. Duplicates were removed in Zotero, automatically and by hand.

Titles and abstracts were screened by two independent researchers (AC, JS). Two additional researchers (JG, MLH) independently screened 50% of all articles to ensure consistency with eligibility criteria. Full texts were retrieved via our university libraries, ResearchGate, or Google Scholar. When the full texts were not available, we reached out to our professional network to inquire whether researchers from other institutions had access to full texts. Full texts selected for review were evenly divided between two pairs of researchers, who independently screened them against the inclusion criteria. The reason for excluding full texts was recorded. When disagreement occurred, all authors discussed the inclusion or exclusion of the publication. An additional researcher (ME) was available to arbitrate if no consensus could be reached. A total of 10545 records were identified across five electronic databases; after removal of 3710 duplicates, 6835 records were screened on title and abstract, of which 183 were selected for full-text review. Following full-text screening, 16 publications met the eligibility criteria and were included in this review (Figure 1).

**Figure 1:**
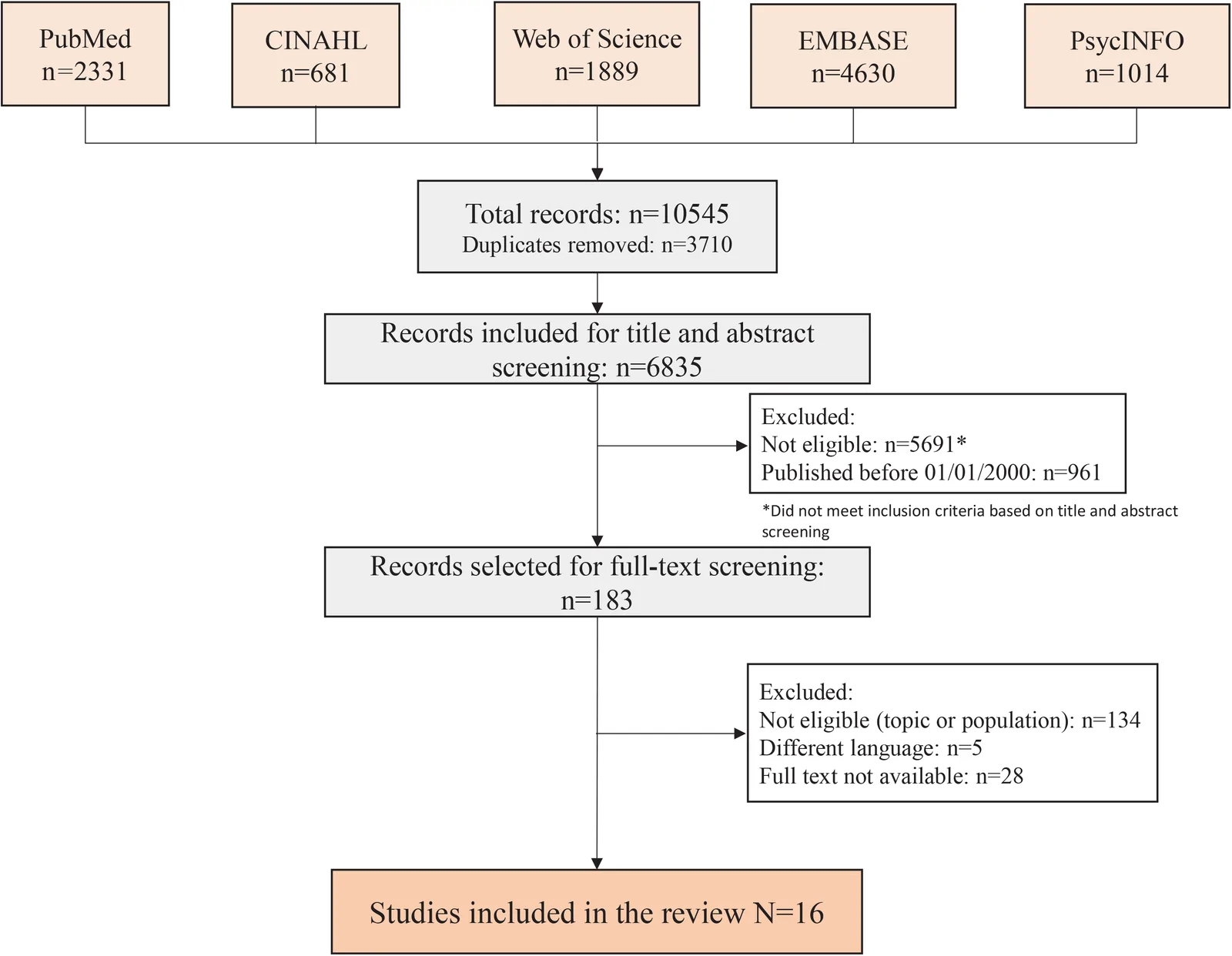
Flow chart of selection of studies

A data extraction tool developed by the researchers was used to obtain relevant data from the results sections of the publications included, capturing the following fields: first author and year of publication, country, study design, population, relevant findings, and screening tools. Data extraction was performed independently by two researchers (AC, JS), with a third researcher (JG) verifying a random sample of 25% of extractions to ensure consistency and accuracy. Discrepancies were resolved through discussion among the research team.

Relevant excerpts were analyzed using inductive thematic analysis, following a six-phase approach^12^: familiarization with the data, generating initial codes, searching for themes, reviewing themes, defining and naming themes, and producing the report. Initial coding was performed independently by two researchers (AC, JS), who each read through the included publications and assigned codes to relevant excerpts without a predefined coding framework, allowing themes to emerge from the data. Coded segments were then compared and discussed between the two coders, and emerging themes were reviewed and refined in consultation with the wider research team (JG, MLH) until consensus was reached. A narrative synthesis of the identified themes is provided.

Sixteen publications, published between 2000 and 2022, are included (Table 2). Most were conducted in the United Kingdom (n=6) or the United States (n=4). Others were conducted in Australia, Canada, France, Germany, India, and Poland. We included two primary studies, eight reviews, one case study, one synthesis of current evidence and treatment guidelines, one unstructured literature overview, two clinical practice articles, and one case report, totaling sixteen publications.

**Table 2:** Characteristics of publications included in a narrative review on the role of midwives in perinatal anorexia nervosa care (N=16)

*Primary studies*					
*No.*	*Study Year*	*Country*	Objective(s)*	*Study design (methods)*	*Description included studies (number of included studies, if provided)*
1	Bye et al.^20^ 2018	UK	To understand barriers to disclosure and identification of ED in pregnancy and postnatally.	Mixed-methods design (survey (1) and focus groups (2), over 7-month period)	(1) Pregnant and postnatal women with current or past eating disorder (n=101); (2) Student and qualified midwives and health visitors in UK NHS (n=33)
2	Stringer et al.^23^ 2010	UK	To explore experiences of pregnant women with an eating disorder and during the early years of the child’s life, including views on healthcare provision.	Qualitative design (oneto-one, semi-structured interviews)	Women with eating disorder and 16 weeks pregnant, or whose youngest child was two years or younger (n=8)
***Reviews***					
***No.***	***Study Year***	***Country***	Objective(s)*	***Study design (methods)***	***Study population (sample size, if provided)***
3	Dutta et al.^27^ 2022	India	To critically analyze and discuss the association of prenatal and postnatal factors that lead to different eating disorders in mother and child.	Unstructured narrative review of academic literature (PubMed, Google Scholar)	‘Published and standard articles’ (n=38)
4	Letranchant et al.^15^ 2022	France	To analyze several specific aspects that women with anorexia nervosa either intend to undergo fertility treatment or become pregnant.	Unstructured overview of peer-reviewed literature (no methods reported)	Infertility women with anorexia nervosa and pregnant women with anorexia nervosa
5	Mitchell et al.^16^ 2006	USA	To address nature and prevalence of eating disorders and the management during pregnancy.	Unstructured overview of peer-reviewed literature (no methods reported)	Pregnant women with eating disorders
6	Nickols-Richardson et al.^17^ 2008	USA	To highlight current evidence on pregnancy and eating disorders	Unstructured overview of peer-reviewed literature (Book chapter)	Pregnant women with anorexia nervosa
7	Pan et al.^22^ 2022	Australia	To explore pregnancy complications in women with active anorexia, including maternal, fetal, and neonatal complications.	Systematic review including primary studies and case studies (PubMed, PsycINFO, CINAHL, SCOPUS)	Studies (n=21) including women with active anorexia nervosa during pregnancy
8	Paslakis et al.^19^ 2019	Canada	To provide specific clinical recommendations on clinical management for gynecologists and fertility specialists on women with eating disorders	Unstructured overview of peer-reviewed literature (no methods reported)	Women who either are pregnant or wish to conceive with eating disorder
9	Tierney et al.^25^ 2013	UK	To examine what is known about women’s experiences of receiving health care when pregnant with an eating disorder and which support they need.	Systematic review (ASSIA, British Nursing Index, CINAHL, Embase, Medline, MIDIRS, PsycINFO, SCOPUS)	Qualitative studies (n=7) including perspectives of women with or self-reporting some form of anorexia nervosa in relation to being pregnant (n=49)
10	Franko and Spurrell^18^ 2000	USA	To examine potential interactions between pregnancy and eating disorders, alert obstetric professionals to warning signs of eating disorders and suggest a simple state-of-the-art assessment strategy and offer treatment guidelines for pregnant women with eating disorders.	Opinion paper, including unstructured review of peer-reviewed literature (no methods reported)	Women with ED in pregnancy (not defined)
11	Cantrell et al.^14^ 2009	USA	To explore risks associated with eating disorders in pregnancy, signs and symptoms of these conditions, and the screening and management of eating disorders in the antepartum setting.	Case study (based on observations from clinical rounds and academic literature)	Case study: one pregnant woman with history of eating disorder
12	Dorsam et al.^28^ 2022	Germany	To synthesize current evidence, treatment guidelines, and data on the diagnostic accuracy for screening for EDs in the antenatal period.	Synthesis of current evidence, treatment guidelines and data on diagnostic accuracy for screening.	Studies (n=3), including pregnant women with eating disorders in antenatal period (not specified)
13	Mandera et al.^21^ 2019	Poland	To review literature on pregorexia (eating disorder which involves self-starvation and/or inducing vomiting during pregnancy).	Unstructured overview of peer-reviewed literature (no methods reported)	Women with first-time pregorexia that occurs during pregnancy (not specified)
14	Stringer et al.^24^ 2019	UK	To increase midwives’ knowledge, skills and confidence about eating disorders with women who are pregnant and how to recognize early signs.	Clinical practice (observation and unstructured peerreviewed literature review)	Pregnant women with eating disorders (not specified)
15	Virgo^26^ 2022	UK	To provide advice for midwives to better support those with a diagnosis or history of eating disorders.	Case report (self-reported)	One pregnant woman with eating disorder
16	Ward et al.^29^ 2008	UK	To share clinical practice experience in women with eating disorders in pregnancy.	Clinical practice article	Women with active anorexia nervosa/eating disorder during perinatal period (not specified)

AN: anorexia nervosa. BED: binge eating disorder. BN: bulimia nervosa. ED: eating disorder. EDNOS: eating disorder not otherwise specified. GP: general practitioner.

* As reported in the publication.

## DEVELOPMENTS

We have identified four key themes related to the midwife’s (potential) role in perinatal care for women with anorexia nervosa and challenges they might encounter.

### Recognizing signs, early and continuous screening

Even though publications stipulate that early identification of an eating disorder (history) is crucial for optimizing care for women during the perinatal period and preventing complications^14-26^, eating disorders are generally underdiagnosed. Patients tend not to voluntarily disclose their eating disorder to healthcare professionals^15,18-20,23,25,27^, due to stigma, shame, lack of opportunity, and a preference for self-management, or perceived lack of empathy from midwives^23^.

Midwives generally do not ask their patients about lifetime eating disorder diagnoses^15,19,23,25^. However, most publications emphasize the need for routine screening for anorexia nervosa during antenatal care, and midwives play a crucial role^14-17,27,28^. However, midwives often lack the confidence and knowledge to identify and address eating disorders and therefore are at risk of being focused on clinical tasks, not giving enough thought to the woman’s weight and eating patterns, even though this should be a top priority, especially if the patient has a history of eating disorders^25^. According to the literature, this is due to a lack of awareness and alertness in identifying signs or knowledge of potential screening tools^19^. There is a lack of knowledge about potential warning signs, such as insufficient weight gain, electrolyte imbalances, and symptoms of malnutrition or dehydration^16,18,27,29^. For instance, Franko et al.^18^ indicate that lack of weight gain in two consecutive visits in the second trimester, a history of an eating disorder, and hyperemesis gravidarum are warning signs. Additionally, Ward et al.^29^ identified other warning signs such as low BMI, concern about weight but not being overweight, menstrual disturbances or amenorrhea, gastrointestinal symptoms, physical signs of starvation or repeated vomiting, and psychological problems.

Screening can be done by asking open-ended questions about food habits and rules, laxative and diuretic use, exercise, medicines for thyroid diseases, and slimming tablets^29^. For example: ‘Do you think you have an eating problem? Do you worry excessively about your weight?’ or asking open-ended questions about the use of herbal supplements, caffeine, and laxatives, which are often used by people with eating disorders^24^. Next to open-ended questions, several screening tools are available: the five questions about Sick, Control, One, Food and Fat (SCOFF) questionnaire^14-16,19,28^, the Eating Disorder Examination Questionnaire (EDE-Q)^14,16,27,28^, the Eating Disorder Examination (EDE)^17,18,28^, the Eating Attitudes Test (EAT-8)^15,19,28^, the Eating Disorder Inventory-2 (EDI-2)^28^, and the Disordered Eating Behavior Scale (DEBS)^28^. These tools can assist in fast and efficient screening for eating disorders, including AN, during antenatal care. When a positive score is found, this should be an indication for referral and diagnostic assessment by a specialist in eating disorders^16^. The SCOFF questionnaire was the most commonly used^14-16,19,28^ and includes five yes/no questions that can be completed in 30 seconds. A score of two or more is considered indicative of an ED, though it is not a diagnostic for non-specialists. This tool is widely validated for screening purposes. However, SCOFF items may overlap with typical pregnancy symptoms, potentially leading to high rates of false positives or false negatives. The National Eating Disorders Collaboration (NEDC) recommends its use during pregnancy, emphasizing the importance of considering weight and BMI, particularly when the screening results are negative.

It is necessary to pay sufficient attention to the eating disorder during every consultation^29^. This includes monitoring vital signs, weight gain and nutritional status, which are crucial^14,16-19,21,22,24-29^. In addition, it is best to have a physical examination during each consultation. The following abnormal values may be identified: hypotension, bradycardia, low body temperature, low body fat, hair loss, and symptoms of anemia. Ketones should be checked at every visit, indicating dehydration and/or starvation^14,17,28^. It is recommended to ask the patient if she wants to know her weight and what type of feedback would be helpful to her^14,17-19,21,23-26,29^. It can be helpful to suggest a blind weigh-in and avoid announcing the BMI out loud in the room.

### Continuity of care, tailored to individual needs and wishes

Continuity of care is important^14,16-18,21,23-29^, ensuring follow-up from the start of antenatal care until at least six months in the postpartum period^15^. Although to a less extent involved due to the highly specialized care, midwives are seen as the key professionals to continuously support pregnant women with eating disorders^14,16,22,25,29^. During pregnancy, they are often the key caretaker, which can promote openness and early intervention^22-24,29^. This relationship of trust may already start during the preconception period^24^. More frequent midwifery follow-ups could be beneficial for the early recognition of maternal complications, the general well-being of the mother and child, and ensuring adequate feeding of the child^22^ – thereby improving pregnancy outcomes for the mother and the development of the child^16^. Also, the postpartum period, in which primarily the midwife is involved, is identified as a period full of changes and might create opportunities for other support, such as early screening of mental health^24,29^, since women with eating disorders have an increased risk of developing postpartum depression. Therefore, it is essential to provide information about this risk during the antenatal period^14,24^. Indirectly, there is also an opportunity to influence a possible next pregnancy^24^. Such preconception advice could include appropriate vitamin supplementation and advising them on healthy levels of exercise before conceiving again^24,29^, decreasing the use of laxatives, appetite suppressants, or diuretics, which are not safe in pregnancy^29^.

It is mentioned that the frequency of care and consultations should depend on the severity of the disorder. An increased number of consultations is desirable, in which vital signs and nutritional status are evaluated to minimize health risks to mother and child^14,18,22,23,29^. The duration of a consultation and length of care should also be adjusted accordingly^29^.

### Building a trusted relationship

A key element in building a trusted relationship is providing non-judgmental support, which should be tailored to the unique needs of each woman^14,16-18,21,23-29^. Building on the relationship of trust, the midwife holds a unique position to gain insight into the specific needs and wishes of the mother^22-24,29^. This requires compassionate and thoughtful care, free from personal judgment, ensuring that women feel safe and respected throughout their care^18,23^. Understanding the woman’s triggers and fears is essential to creating a concrete action plan, with clear goals set for each consultation^14,23^. Midwives should focus on guidance, ensuring that women know what to expect during each consultation^14,23^.

### Nutrition counseling and breastfeeding support

Although five publications indicate that there is currently poor professional awareness and accurate knowledge about providing nutritional advice^15,19,23,25,26^, most publications recommend individualized nutritional counseling and ongoing education about the benefits, both during pregnancy and after childbirth^14,16-19,21,22,24-29^. Prenatal counseling should focus on providing nutritional advice to support a healthy pregnancy. This includes guidance on body changes, cravings, and the management of hyperemesis gravidarum^29^. Despite weight control being recommended, it is unclear, however, what rate of gestational weight gain would be sufficient to sustain a healthy pregnancy^22^. Also, women with eating disorders tend to be more likely to experience breastfeeding challenges^16,21,24,27,29^. It is suggested that encouraging women to breastfeed might help them achieve a normal weight^24^. Postnatal counseling can support healthy eating habits and address any nutritional issues that may arise during breastfeeding.

### Need for education and training of midwives

Seven articles highlight the need for education and training of midwives regarding AN^15,19,20,23,25,26,28^. Two studies suggest education and training might reduce the stigma surrounding eating disorders, potentially leading to better reporting and treatment of symptoms by women^25,28^. Studies recommend training for all professionals involved in perinatal care to increase awareness and alertness in identifying eating disorder^19^, in midwives education must focus on recognizing potential warning signs^16,18,27,29^ and potential risks and pathologies (e.g. pre-eclampsia, preterm birth, low weight gain, and postpartum depression). They can learn about the importance of ongoing monitoring; also after birth, and screening for additional pathologies^16,22,23,27,29^. They also need to be aware of potential referral pathways to specialized care, if necessary.

Healthcare professionals also lack the appropriate skills to communicate carefully about it^23,25,26^. In the study by Tierney et al.^25^, a study participant testified:

*‘It was all written down there in the file, but there was no reaction … I was never asked any questions about it. No one ever mentioned it.’*

Authors, therefore, stipulated the need for a certain openness towards talking about it^26^*.* Stringer et al.^24^ described staff being perceived as thoughtless in their use of language. Comments from midwives such as: ‘you look well, you are healthier than the overweight mums’ are apparent but do not help. Tierney et al.^25^ recommend developing educational interventions aimed at training communication skills that enable midwives to discuss the topic of eating and weight control in a safe and supportive manner.

### An interprofessional approach

Treatment of an eating disorder, especially in pregnancy, requires an interprofessional team approach14,15,17,18,21-24,27,29, involving gynecologists, midwives, dietitians, psychologists, and psychiatrists. This approach facilitates the early recognition of potential complications and promotes healthy nutrition and care for both the mother and the child^16,17,21,22,24,27,29^. Women who disclosed their eating disorder often felt labeled as ‘high-risk’, and studies have shown they are more likely to receive more hospital-based or specialist care, resulting in receiving a lot of care from multiple physicians^24,29^. To ensure holistic, well-coordinated management and continuity of care of eating disorders in pregnant women, it is therefore important that healthcare providers work together effectively, as two studies mention24,29.

### Implications

This review summarizes the peer-reviewed academic literature that might inform the role of midwives in providing perinatal care to women with anorexia nervosa during the perinatal period. We included sixteen publications published after 2000. In the available literature, the role of a midwife is considered in: 1) recognizing signs, early and continuous screening; 2) continuity of care, tailored to individual needs and wishes; 3) building a trusted relationship; and 4) nutrition counseling and breastfeeding support. However, considerable challenges reported might hinder the uptake of this role, and the need for an interprofessional approach and specialist referral, if needed, to ensure continuity of care and careful treatment was a central theme.

First, despite the clear consensus that early and continuous screening of eating disorders is crucial for optimal perinatal care in this population, midwives and other healthcare professionals often do not ask their patients about lifetime eating diagnoses. The lack of routine screening and proactive inquiry contributes to suboptimal care for those women^17^ and can negatively impact factors that are important for the outcome. These include psychological, social, and physical health outcomes, psychopathology, and treatment-seeking behaviors^30^. It can lead to underdiagnosis, exacerbated by patients’ reluctance to disclose their eating disorder due to stigma, lack of opportunity, and a preference for self-management^18,20^. Facilitating client self-disclosure is found to be essential to therapeutic effectiveness^31^. As discussed, various screening tools are available, though their effectiveness in this population warrants further evaluation, and it is almost self-evident that open communication is key while considering careful use of language. The healthcare professional may unintentionally comment or provide words of advice to a patient struggling with an eating disorder that can trigger or perpetuate the disorder. Education in communication with this specific population is valuable. Screening is important, especially since publications report that in various countries, there is no shared patient file, and disclosure is up to the mother herself. It is, therefore, important that midwives provide an atmosphere in which the patient feels safe enough to disclose this^31^. Participants in the publications reported that addressing this issue is challenging, and therefore, they often resort to using screening tools that indirectly introduce the topic. Also, next to screening for eating disorders, continuous screening and treatment for other mental health issues, including postpartum depression, to which these women are at high risk, is essential. The NICE guideline on ‘eating disorders’ (2020) states that ‘An interdisciplinary approach, including mental health care, is indispensable’^19^.

Second, continuity of care was a central theme in almost all publications. As outlined in the results, monitoring practices and consultation frequency should be tailored to the severity of the disorder^22^. This finding in our review is consistent with the practice NICE guideline on ‘eating disorders: recognition and treatment’ ([NG69], 2020). The guideline provided by the Australian National Eating Disorders Collaboration (NEDC), is the only guideline that explicitly considers the effects of an eating disorder during pregnancy. They recommend continued screening for eating disorders in the perinatal period (National Eating Disorders Collaboration, 2015)^28^. The available guidelines on eating disorders do not consider the role of a midwife, despite mentioning other healthcare disciplines. A specialist referral often remains necessary^32^. However, we did not find clear guidance on when to refer and at what points a follow-up or longer consultation is needed. The midwife by some publications was seen to be primarily responsible for maintaining continuity, making referrals when necessary, and ensuring that both she and the woman retain control over the perinatal care process^11^. Effective interprofessional care ensures that while specialists are involved when needed, the midwife can still maintain oversight of the care process, fostering a more seamless experience for the patient^11^. A call for heuristics or a decisional flowchart for referral might be helpful.

Third, midwives, due to their ongoing role throughout pregnancy, birth, and postpartum, are well-placed to ensure continuous care. Building a trusted relationship with patients promotes openness and early intervention, which is crucial for early recognition of complications and ensuring the well-being of both mother and child^33,34^. As continuity of care is critical for managing eating disorders, even more so during pregnancy, a midwifery-led approach can be valuable. The effectiveness of this model has been tested across the world and has been shown to have a significant positive impact on improving various maternal and child outcomes^11^. However, various barriers still inhibit the implementation of this model (i.e. lack of infrastructure, and role division because of the many disciplines involved, especially in the case of the topic at hand and resources)^35^. Understanding the patient’s unique triggers and fears allows for a midwife to develop a concrete plan of action and clear goals for each consultation – at least, that is what we learned from a case report^26^. However, since in Belgium, there is no additional fee schedule for midwives consulting with patients who have an eating disorder, we expect that some may be reluctant to engage in longer consultations.

Fourth, although midwives can play a crucial role in facilitating breastfeeding, it is unclear from the literature that we assessed whether it is the role of the midwife to provide nutritional counseling and whether they are trained properly to do so. As noted, midwives often lack confidence in nutritional counseling. A collaborative approach between midwifery bodies, nutrition (i.e. dietitians), and education experts might provide an effective way forward^36^. Additional expertise, however, on cravings and specifics to eating disorders is necessary. Weighing was considered by many publications when nutrition was discussed. We question whether this is helpful since there is no specific guideline on what ‘sufficient’ weight gain in pregnancy is. Recent guidelines state that appropriate gestational weight gain should be personalized^37^. Since there are no clear recommendations on how much weight needs to be gained for a healthy pregnancy^22^, should it be part of routine management in this population? This question highlights an area that would benefit from further research, as it would be interesting to explore how integrating specific weight gain guidelines could impact the management of these women. Also, breastfeeding support remains an underexplored area in this population, although a midwife might be well-placed to guide this.

The need for interprofessional collaboration is recognized in all referenced publications. However, when to refer and how to collaborate effectively is an ongoing challenge. Clinical pathways or suggestions for interprofessional management are missing in the literature^19^. The currently available literature shows a significant need for education and training of midwives in anorexia nervosa. Training should cover recognizing and screening eating disorders, understanding associated risks and complications, careful communication and how to collaborate and refer. In Belgium, the Centre of Expertise for Eating Disorders (‘Eetexpert’ in Dutch) might play a valuable role in providing dedicated training to healthcare professionals involved (https://eetexpert.be/).

We found no evidence of the effectiveness of specifically developed interventions targeting eating disorders in pregnancy. Controlled trials are needed to evaluate the effect of treatments for pregnant women with eating disorders. There is a vast amount of literature describing effective treatments for people with eating disorders (i.e. cognitive therapy or family therapy). These have not yet been tested in pregnant women. Based on our findings, potential areas for further research might include the value of ‘reference midwives’ who have specific knowledge about eating disorders and can provide advice to colleagues in complex cases, the importance of making timely referrals to the appropriate agencies, or ways to improve communication about eating disorders during pregnancy.

### Strengths and limitations

The strengths of this review are highlighted by its comprehensive methodology, ensuring a thorough and replicable process. We consulted five major electronic databases and screened reference lists, allowing for a wide capture of relevant literature. The review also benefited from the support of a librarian in developing the search strategy and a resonance group to refine the research question and approach. Rigorous study screening and selection involving multiple independent researchers minimizes bias and ensures consistency. The protocol was pre-registered.

The review has some limitations. First, there might be a language bias in our study selection. Second and most importantly, the absence of a quality assessment for the studies included affects the robustness of our reporting, although this is not required for a narrative review. Third, although the review includes a broad range of eligibility criteria, we did not engage with grey literature, potentially omitting significant practice guidelines that might have contributed to answering our research questions. In addition, a key limitation of this review is the predominance of non-primary evidence sources (narrative reviews, clinical practice articles, case reports, and opinion papers), with only two primary studies identified. Findings should therefore be interpreted with caution, and future primary research is needed to build a more robust evidence base for midwifery practice in perinatal anorexia nervosa care. Fourth, although we discussed various tools that can be used for screening, we have not assessed their validity. A challenge was the terminology used in the articles: we excluded studies that did not mention midwives specifically and, therefore, might have missed results relevant to and where a midwife was referred to as a nurse or vice versa. Finally, despite the thorough search strategy, the inability to find full texts for twenty-eight articles means that potentially relevant information could have been left out.

## CONCLUSION

This narrative review suggests that midwives may have a meaningful role in supporting women with anorexia nervosa during the perinatal period, particularly in the areas of screening, continuity of care, building a trusted relationship, and breastfeeding support. However, given that the available evidence is largely based on non-primary sources – including reviews, clinical practice articles, case reports, and opinion papers – these findings should be interpreted with caution. Challenges reported in the literature, such as inconsistent screening practices, limited interprofessional collaboration, insufficient midwifery education on eating disorders, and difficulties facilitating self-disclosure, suggest that there are considerable barriers to optimal care, though the extent and generalizability of these barriers remain unclear. A notable gap in the literature is the absence of clear clinical guidelines addressing the midwife’s role in this context, and no effectiveness studies of midwife-led interventions were identified. Future research should prioritize primary studies – including qualitative work exploring the experiences of women and midwives, and controlled trials evaluating specific interventions; as well as the development of evidence-based guidelines and training programs to better define and support the midwife’s role in perinatal anorexia nervosa care.

## Supplementary Material



## Data Availability

The pseudonymized data supporting this research are available from the authors on reasonable request.
