# Supplementary material for: The role of a midwife during perinatal anorexia nervosa care: A narrative review
Source: Eur J Midwifery. 2026 Oct 9;10:10.18332/ejm/236730. doi: 10.18332/ejm/236730 (PMC13653360; doi:10.18332/ejm/236730)
Supplement: Supplementary file 1 [file EJM-10-46-s1.pdf]

EUROPEAN JOURNAL
OF MIDWIFERY

Supplementary file

© 2026 Steppe J. et al.

DOI:
10.18332/ejm/236730

The content has been provided by the author(s) and has not been reviewed, verified, or endorsed by European Publishing. It may not have undergone peer review. The views, opinions, and recommendations expressed are solely those of the author(s) and do not necessarily reflect the position of European Publishing. European Publishing accepts no responsibility or liability for any consequences arising from the use of, or reliance on, this content.

SUPPLEMENTARY FILE

Supplementary Table 1. Search strings used across five electronic databases for a narrative review on the role of midwives in perinatal anorexia nervosa care (search conducted October 2023 – September 2024)

**Database**	**Search string**
PubMed	(pregnan* OR ‘Pregnancy’[Mesh] OR prenatal OR perinatal OR ‘expectant mother*’ OR ‘pregnant woman*’ OR ‘Pregnant Women’[Mesh] OR antepartum OR ‘Perinatal Care’[Mesh] OR ‘perinatal care’ OR ‘Preconception Care’[Mesh] OR ‘preconception care’ OR preconception OR ‘Prenatal Care’[Mesh] OR ‘prenatal care’ OR ‘Postnatal Care’[Mesh] OR ‘postnatal care’ OR postnatal OR ‘Postpartum Period’[Mesh] OR ‘postpartum period’ OR postpartum OR peripartal) AND (‘anorexia nervosa’ OR anorex* OR ‘eating disorder*’ OR ‘feeding disorder*’ OR ‘Anorexia Nervosa’[Mesh] OR ‘Feeding and Eating Disorders’[Mesh])
Web of Science	(pregnan* OR prenatal OR perinatal OR ‘expectant mother*’ OR ‘pregnant wom*’ OR antepartum OR ‘perinatal care’ OR ‘preconception care’ OR preconception OR ‘prenatal care’ OR ‘postnatal care’ OR postnatal OR ‘postpartum period’ OR postpartum OR peripartal) AND (‘anorexia nervosa’ OR anorex* OR ‘eating disorder*’ OR ‘feeding disorder*’)
Embase	(pregnant OR 'pregnancy'/exp OR pregnancy OR 'prenatal'/exp OR prenatal OR perinatal OR 'expectant mother'/exp OR 'expectant mother' OR 'expectant mothers' OR 'pregnant woman'/exp OR 'pregnant woman' OR 'pregnant women'/exp OR 'pregnant women' OR antepartum OR 'perinatal care'/exp OR 'perinatal care' OR 'preconception care'/exp OR 'preconception care' OR 'preconception'/exp OR preconception OR 'prenatal care'/exp OR 'prenatal care' OR 'postnatal care'/exp OR 'postnatal care' OR postnatal OR 'postpartum period'/exp OR 'postpartum period' OR 'postpartum'/exp OR postpartum OR peripartal) AND ('anorexia nervosa'/exp OR 'anorexia nervosa' OR 'anorexia'/exp OR anorexia OR 'anorexic'/exp OR anorexic OR 'eating disorder'/exp OR 'eating disorder' OR 'eating disorders'/exp OR 'eating

	disorders' OR 'feeding disorder'/exp OR 'feeding disorder' OR 'feeding disorders')
CINAHL	(pregnan* OR prenatal OR perinatal OR ‘expectant mother*’ OR ‘pregnant wom*’ OR antepartum OR ‘perinatal care’ OR ‘preconception care’ OR preconception OR ‘prenatal care’ OR ‘postnatal care’ OR postnatal OR ‘postpartum period’ OR postpartum OR peripartal) AND (‘anorexia nervosa’ OR anorex* OR ‘eating disorder*’ OR ‘feeding disorder*’)
PsychINFO	(pregnan* OR prenatal OR perinatal OR ‘expectant mother*’ OR ‘pregnant wom*’ OR antepartum OR ‘perinatal care’ OR ‘preconception care’ OR preconception OR ‘prenatal care’ OR ‘postnatal care’ OR postnatal OR ‘postpartum period’ OR postpartum OR peripartal) AND (‘anorexia nervosa’ OR anorex* OR ‘eating disorder*’ OR ‘feeding disorder*’)
